# Aripiprazole disrupts cellular synchrony in the suprachiasmatic nucleus and enhances entrainment to environmental light–dark cycles in mice

**DOI:** 10.3389/fnins.2023.1201137

**Published:** 2023-08-09

**Authors:** Ruoshi Li, Kosaku Masuda, Daisuke Ono, Takashi Kanbayashi, Arisa Hirano, Takeshi Sakurai

**Affiliations:** ^1^International Institute for Integrative Sleep Medicine (WPI-IIIS), University of Tsukuba, Tsukuba, Japan; ^2^Institute of Medicine, University of Tsukuba, Tsukuba, Japan; ^3^Department of Neuroscience II, Research Institute of Environmental Medicine, Nagoya University, Nagoya, Japan; ^4^Ibaraki Prefectural Medical Center of Psychiatry, Kasama, Japan

**Keywords:** aripiprazole, desynchronization, entrainment, SCN, sleep disorder, circadian rhythm, jet-lag

## Abstract

Many patients with psychiatric conditions, such as bipolar disorder and major depressive disorder, frequently experience disruptions in their sleep–wake cycles. Several case studies and clinical trials have shown that the administration of aripiprazole, a commonly prescribed antipsychotic drug, alleviates the symptoms of circadian sleep disorders in these patients. This improvement may be attributed to the effects of aripiprazole on the circadian central clock, specifically the hypothalamic suprachiasmatic nucleus (SCN), which regulates various circadian physiological rhythms, including the sleep–wake cycle, in mammals. To examine whether aripiprazole facilitates adaptation to changes in the light–dark cycle, we orally administered aripiprazole to mice and subjected them to jet-lag experiments. Mice receiving aripiprazole were more rapidly entrained to 6 h advanced light–dark cycles. Moreover, we examined the effect of aripiprazole on the cellular rhythms of SCN slice cultures and found that aripiprazole disrupted cellular synchronization in the SCN, thereby accelerating the damping of the SCN rhythm at the population level. Adenosine 3′5’ monophosphate (cAMP) assay using a bioluminescence indicator revealed that intracellular cAMP level in the SCN increased following aripiprazole treatment. However, this increase was blocked by pre-treatment with the serotonin 1A receptor (5-HT_1A_R) antagonist. Based on these findings, we propose that aripiprazole modulates intracellular signaling, including 5-HT_1A_R-mediated cAMP signaling, and desynchronizes SCN neurons, ultimately leading to enhanced entrainment to phase advanced light–dark cycles in mice. These findings indicate that the improvement in sleep symptoms reported in patients with psychiatric disorders receiving aripiprazole may be due to modulation of the circadian clock. Our study provides novel insights into the potential clinical applications of aripiprazole in patients with various circadian sleep disorders.

## 1. Introduction

Aripiprazole is an atypical antipsychotic drug commonly used to treat schizophrenia and bipolar disorder (Kern et al., 2006; Wang et al., 2012). It acts on various G protein-coupled receptors (GPCRs) and functions as a partial agonist of the dopamine D2 receptor (D2R) and serotonin 1A receptor (5-HT_1A_R) while simultaneously functioning as an antagonist of the 5-HT_2A_ receptor (Kikuchi et al., 1995; Shapiro et al., 2003). Aripiprazole exerts diverse effects by modulating various subtypes of dopamine and serotonin receptors, including 5-HT_2B_R, 5-HT_2C_R, 5-HT_6_R, 5-HT_7_R, D3R, and D4R (Shapiro et al., 2003), rendering its mechanism of action highly complex.

Recent studies have reported that aripiprazole administration improves sleep disorder symptoms in humans, particularly those associated with rhythmic abnormalities (Takaki and Ujike, 2014; Matsui et al., 2017; Tashiro, 2017; Omori et al., 2018; Imanishi et al., 2021; Konishi et al., 2022). In a case report, a female patient with delayed sleep-phase syndrome (DSPS) could entrain to environmental light–dark cycles following aripiprazole treatment, which improved daytime sleepiness and depressive mood (Takaki and Ujike, 2014). In another case report, a male patient with concomitant bipolar and sleep disorders exhibited improved symptoms, including reduced sleep splitting and delayed sleep offset, after resuming aripiprazole treatment (Tashiro, 2017). In a female patient with major depressive disorder and a non-24-h sleep–wake rhythm disorder, aripiprazole treatment aligned sleep–wake cycle with the environmental light–dark cycle (Matsui et al., 2017). Additionally, aripiprazole reduced prolonged nocturnal sleep time in a teenage female with idiopathic hypersomnia (Imanishi et al., 2021). In a case trial involving 12 patients with DSPS who were treated with aripiprazole, sleep onset and offset as well as sleep midpoint were significantly advanced, and sleep duration was significantly shortened (Omori et al., 2018). Moreover, aripiprazole, when administered in combination with blue light, has been shown to be effective in treating wake-up difficulties in young individuals, further suggesting the efficacy of the drug in improving adaptation to the external light–dark cycle (Konishi et al., 2022). Taken together, these findings suggest that aripiprazole may serve as a potential therapeutic agent for sleep disorders, particularly circadian rhythm sleep disorders, such as DSPS. However, mechanism underlying the aripiprazole-mediated improvement of circadian sleep disorder symptoms remains unclear.

The circadian clock produces a significant impact on the sleep–wake cycle. In mammals, the hypothalamic suprachiasmatic nucleus (SCN) serves as the central clock (Weaver, 1998), and neuronal coupling among SCN neurons generates robust circadian oscillations that persist even under constant conditions without entrainment cues (e.g., light–dark cycle). SCN neurons express several types of monoamine receptors, which may be the pharmacological targets of aripiprazole (Wright et al., 1995; Rivkees and Lachowicz, 1997; Pronina et al., 2020). In particular, D1R, D2R, and D5R are expressed in SCN neurons (Rivkees and Lachowicz, 1997; Pronina et al., 2020). Among these, D2R is a well-known target of aripiprazole; however, its physiological significance in circadian behavioral regulation remains unknown. In contrast, the activation of 5-HT_1A_R, another receptor expressed in the SCN, by a selective agonist resulted in accelerated entrainment to new light–dark cycles in mice (Moriya et al., 1998; Takahashi et al., 2002). 5-HT_2C_R is also expressed in the SCN (Pickard and Rea, 1997). Studies have shown that *in vitro* application of a 5-HT_2C_R agonist increases *Per1* and *Per2* expression in the SCN (Varcoe et al., 2003; Varcoe and Kennaway, 2008). Additionally, *in vivo* administration of the 5-HT_2C_R agonist induced a phase shift of melatonin level and core body temperature rhythms (Kennaway and Moyer, 1998). 5-HT_7_R is also expressed in the SCN, and *in vivo* administration of a selective 5-HT_7_ R antagonist in combination with a selective serotonin reuptake inhibitor has been reported to induce a phase shift (Westrich et al., 2013). Thus, given the notable efficacy of aripiprazole in treating circadian rhythm sleep disorders, this drug may directly affect the SCN by modulating monoamine functions. If this medication promotes entrainment to external light–dark cycles, it may lead to an earlier onset and offset of sleep as well as decrease prolonged sleep time in patients with DSPS.

Here, we demonstrate that mice orally administered with aripiprazole exhibited faster entrainment to a phase advanced external light–dark cycle. We further found that aripiprazole desynchronized cellular rhythms in SCN neurons, possibly through receptors in the SCN, such as the 5-HT_1A_R, that are involved in cAMP signaling, thereby accelerating the damping of PERIOD2 (PER2) oscillation amplitudes at the population level. These observations imply that aripiprazole affects the circadian master clock, leading to better adjustment to environmental photic stimuli and subsequently improving the symptoms of patients with DSPS.

## 2. Materials and methods

### 2.1. Animals

All behavioral experiments were performed using 10–20-week-old male wild-type C57BL/6 J mice purchased from CLEA Japan Inc. To monitor the bioluminescence of the fused proteins of PER2 and LUCIFERASE (PER2::LUC) in slice SCN cultures, we used 6–20-week-old male or female *mPer2^Luc^* knock-in (KI) homozygous mice purchased from Jackson Laboratory (B6.129S6-Per2tm1Jt/J, strain #006852; Yoo et al., 2004). For adenosine 3′5’ monophosphate (cAMP) assay, neonatal (3–7-day-old) male or female C57BL/6 J wild-type mice were used. The mice were maintained under a 12/12 h light/dark (LD) cycle (except for the assessment of free-running behavior in constant darkness) in a temperature- and humidity-controlled room and fed *ad libitum*. All experimental procedures were approved by the Animal Experiment and Use Committee of the University of Tsukuba and were performed in accordance with the NIH guidelines.

### 2.2. Activity recording and aripiprazole administration

Animal activity was measured using running wheels. Mice were housed individually in cages equipped with running wheels (MELQUEST, Japan). The cages were maintained in light-tight chambers illuminated with white light-emitting diodes (LEDs; 100 lx). Wheel running activities in the LD conditions were recorded in 1 min bins using ClockLab Data Collection (Actimetrics, USA). The animals were first entrained to a 12/12 h LD cycle (9:00 AM lights on, 9:00 PM lights off) for at least 10 days, during which they were administered 1% arabic gum (FUJIFILM Wako Pure Chemical Corporation; Cat #016–00025) through drinking water to measure daily water intake, as described previously (Segnitz et al., 2009, 2011; Peselmann et al., 2013). Aripiprazole suspensions (Tokyo Chemical Industry Co., Ltd.; Cat #129722–12-9) were prepared in 5% arabic gum and diluted in drinking water to the final concentration of 1% arabic gum. The control animals received drinking water containing 1% arabic gum. Aripiprazole doses of 12.5, 20, and 40 mg·kg^−1^·day^−1^ were adjusted to the daily water intake and body weight of each mouse. Water bottles were changed twice a week and water consumption for each mouse was measured every time to monitor the amount of aripiprazole taken. Afterward, the LD cycle was 6 h phase advanced (3:00 AM lights on, 3:00 PM lights off). The mice were maintained under the shifted LD cycle for at least 10 days to evaluate the number of days required for re-entrainment. The onset of wheel-running activity in each mouse was determined by a blinded investigator using ClockLab Analysis 6 (Actimetrics, USA); the time point with the largest difference in activity level 6 h before and after the point was determined to be the onset. When the onset was difficult to determine, the onset defined by ClockLab Analysis 6 was prioritized. The 50% phase shift value (PS50) was calculated by fitting the onsets for 14 days (4 days before and 10 days after jet-lag) to a sigmoidal dose–response curve with a variable slope, Y = Bottom + (Top – Bottom)/(1 + 10^(log PS50 – X) Hillslope^), where X is the log of the dose, Y is the response, Top and Bottom are the plateaus of Y, Hillslope is the slope factor. Data of one mouse that did not fit the sigmoidal dose–response curve (negative R squared value) was excluded. To measure the free-running rhythm, mice administered with 0, 20, and 40 mg·kg^−1^·day^−1^ aripiprazole were kept in constant darkness and their wheel-running activity was recorded for 2 weeks. The circadian period and Qp value (a power of chi-square periodogram used as an indicator of rhythm robustness) were calculated using ClockLab Analysis 6.

### 2.3. Analyzing the effect of aripiprazole on photic input to the SCN

Ten mice were administered with 40 mg·kg^−1^·day^−1^ aripiprazole in 1% arabic gum (made with the aforementioned procedure) for 2 weeks while another 10 were administered with only 1% arabic gum as control. All mice were housed in home cages in 12/12 h LD condition. After two-weeks of aripiprazole administration, 5 mice from each condition (control or 40 mg·kg^−1^·day^−1^ aripiprazole) were given a 30 min light pulse from zeitgeber times (ZT) 21.5 to ZT22. These mice were immediately deeply anesthetized with avertin (2% 2,2,2-Tribromoethanol [SIGMA-ALDRICH; Cat#T48402], 1.2% 2-Methyl-2-butanol [FUJIFILM Wako Pure Chemical Corporation; Cat#010–03703], 8% Ethanol [FUJIFILM Wako Pure Chemical Corporation; Cat#057–00456] in Phosphate Buffered Saline [PBS] [Nacalai Tesque; Cat# 27575–31]), and then perfused transcardially with PBS, followed by 4% paraformaldehyde (PFA, Nacalai tesque; Cat#02890–45) in PBS. Brains were removed and postfixed overnight in 4% PFA at 4°C and transferred to 20% sucrose (FUJIFILM Wako Pure Chemical Corporation; Cat#196–00015)/PBS. After overnight incubation at 4°C, brains were frozen with liquid nitrogen. Coronal brain sections were sliced at 25 μm using a cryostat (Leica Biosystems). The other 5 mice from the control and 40 mg·kg^−1^·day^−1^ aripiprazole administered groups were not given the 30 min light pulse and their brains were sampled at ZT22 following the procedure mentioned above.

Serial brain sections were collected in PBS and incubated in 24-well plates with blocking buffer (0.2 v/v% Triton X-100 [MP Biomedicals; Cat#807426], 3% pH5.2 Albumin, Bovine Serum [BSA, Nacalai tesque; Cat#01863–48]/PBS) for 30 min. The sections were incubated with the primary antibody, rabbit anti-c-FOS antibody (1:1000; EnCor Biotechnology Inc.; Cat# RPCA-c-FOS, RRID: AB_2572236) in the blocking buffer at 4°C overnight. The sections were rinsed two times for 10 min each with PBS and once for 10 min with blocking buffer followed by secondary antibody treatment with Alexa Fluor 488 donkey anti-rabbit IgG antibody (1:1000; Thermo Fisher Scientific; Cat#A-21206, RRID: AB_2535792) at 4°C overnight. Sections were counterstained with 1 mg·mL^−1^ DAPI solution (Dojindo; Cat#D523) diluted by PBS at a ratio of 1:1000. After incubation, sections were rinsed three times for 10 min each with PBS, mounted, and coverslipped. Images were obtained with a confocal microscope (TCS SP8; Leica Microsystems).

### 2.4. PER2::LUC bioluminescence recording

6–20-week-old male or female *mPer2^Luc^* homozygous mice were sacrificed by spinal dislocation at ZT5–10. Their brains were rapidly removed and placed in chilled 1 × Hanks’ balanced salt solution (Gibco Life Technologies Corporation; Cat #14025–092) on ice. Coronal sections (150 μm) including the SCN were prepared using a vibratome (VT1200; Leica Biosystems), and a triangular area around 2 mm^2^ including the SCN was cut out from each section. For examining the effect of different doses of aripiprazole on SCN slice cultures, a total of 18 SCN slice cultures were collected from 9 mice, and 3 slices were allocated to each aripiprazole dose group (total of 6 groups). For examining the effect of 20 μM aripiprazole on SCN slice cultures, a total of 16 SCN slice cultures were collected from 7 mice. The slices were individually placed on Millicell Cell Culture Inserts (Merck KGaA; Cat#PICM0RG50) in a 35 mm tissue culture dish (IWAKI AGC Techno Glass; Cat #3000–035) with 1.5 mL of low glucose Dulbecco’s modified Eagle’s medium (Merck KGaA; Cat#D2902) containing 10 mM HEPES (pH 7.0), 3.5 g·L^−1^ d-glucose, 0.5 mM d-luciferin potassium salt (FUJIFILM Wako Pure Chemical Corporation; Cat #126–05116), 2% B-27 supplement (Merck; Cat #17504–044), 35 mg·L^−1^ NaHCO_3_ (FUJIFILM Wako Pure Chemical Corporation; cat #197–01302), and 1% penicillin–streptomycin (10,000 units·mL^−1^ penicillin and 10,000 mg·mL^−1^ streptomycin; Merck; Cat #15140–122). The medium was prepared prior to the experiments. Aripiprazole was dissolved in dimethyl sulfoxide (DMSO; Nacalai Tesque; Cat #13408–64) to prepare a 20 mM stock solution, which was added to the culture medium at the indicated concentrations (see figure legends). For controls, the same amount of DMSO was added to the culture medium. Slice cultures were maintained at 37°C, and bioluminescence was recorded with either luminometer Kronos Dio (AB-2550, ATTO) for 1 min every 9 min for 10 days or live single-cell imaging system Cellgraph (AB-3000B, ATTO) for 29 min at 1 min intervals for 5 days. Data obtained from the Cellgraph were analyzed using Cellgraph Viewer (ATTO) by randomly selecting 20 neurons in each slice. Cells are outlined with circles, temporal locations were adjusted, and bioluminescence per pixel was calculated using CellGraph Viewer (ATTO).

### 2.5. cAMP assay

For SCN slice preparation, neonatal (3–7-day-old) male or female C57BL/6 J wild-type mice were sacrificed, and their brains were removed. SCN slices were collected and cultured as described above (section 2.4), except for the slice thickness, which was 250 μm. Three days after slice preparation, an adeno-associated virus (AAV) vector expressing Okiluc-aCT (*AAV10-hSyn-Okiluc-aCT*; 1.0 × 10^13^ gc·mL^−1^), a recently developed cAMP bioluminescence sensor, was added to the SCN slices, as previously described (Ono et al., 2023). The AAV vector was generated using the AAVpro Helper-free system (Takara Bio Inc.). Briefly, HEK293T cells were co-transfected with the pAAV-hSyn-Okiluc-aCT, pHelper, and pRC plasmid vectors and cultured for 72 h. The AAV vector was extracted using AAVpro extraction solution, according to the manufacturer’s protocol (Takara Bio Inc.; Cat #6235), and viral titer was determined using real-time PCR. The culture medium same as described above (section 2.4) but without 0.5 mM d-luciferin potassium salt, was replaced with fresh medium every 3 days. Ten days after the virus infection, the culture medium was replaced with a medium containing 0.3 mM d-luciferin potassium salt (FUJIFILM Wako Pure Chemical Corporation; Cat #126–05116), and bioluminescence derived from Okiluc-aCT was recorded using Kronos Dio (AB-2550, ATTO) for 1 min every 10 min. 20 μM aripiprazole was added to the medium 24 h after the start of the recording. For the cAMP assay testing the involvement of the 5-HT_1A_R, WAY-100635 maleate (abcam; Cat #ab120550) was added to the medium 24 h after the start of recording. WAY-100635 maleate was dissolved in DMSO to prepare a 20 mM stock solution, which was added to the culture medium at the final concentration of 20 μM. For controls, the same amount of DMSO was added to the culture medium. 20 μM aripiprazole was then added 24 h after the WAY-100635 or DMSO application. Bioluminescent images from Okiluc-aCT were captured using a CCD camera (Cellgraph, AB-3000B, ATTO). Data from slices without bioluminescence signals in the SCN were excluded from analysis. For the cAMP assay with only aripiprazole, bioluminescence signals were normalized to the bioluminescence value immediately before aripiprazole treatment and plotted. For the cAMP assay with WAY-100635, the recorded data were detrended, as the recording days were longer than cAMP assay with only aripiprazole, resulting in damping of the baseline of the bioluminescence signal.

### 2.6. Analysis of bioluminescence rhythms

Let $l_{j}$ be the bioluminescence at the *j*^th^ measurement point. Since bioluminescence data often contain noise, the data that satisfy $\left|l_{j}-l_{j-1}\right|>\left|l_{j+1}-l_{j-1}\right|\times 10$ and $\left|l_{j}-l_{j+1}\right|>\left|l_{j+1}-l_{j-1}\right|\times 10$ were replaced by $\tilde{l_{j}}=\left(l_{j+1}+l_{j-1}\right)/2$ to remove noise.

Normalization was performed based on a previous study (Masuda et al., 2023) as follows:


(1) $$L_{j}=\frac{l_{j}-\bar{l_{j}}}{\bar{l_{j}}},$$

(2) $$\bar{l_{j}}=\frac{1}{2n+1}\overset{2n}{\underset{j=0}{\sum }}l_{k+j-n}$$

where $\bar{l_{j}}$ is the moving average with a 24 h window, $L_{j}$ is the normalized bioluminescence, and n is the number of data points within 12 h (*n* = 24 in the single-cell imaging data and 80 in the population data).

Cosine fitting was performed to calculate period, amplitude, and phase using the least-squares method in Python (lmfit^1^) based on the previous study (Masuda et al., 2023). The period of the fitted curve was limited to 16–32 h. Period and amplitude were obtained via cosine fitting on normalized bioluminescence on the first 3 days. Temporal changes in phase and amplitude at each time point were obtained via cosine fitting on data for 12 h before and after each point (24 h in total). The synchronization rate was calculated using the following equation:


(3) $$R_{j}=\left|\frac{1}{m}\overset{m}{\underset{k=1}{\sum }}e^{i\theta_{j,k}}\right|.$$

where $R_{j}$ is the synchronization rate at *j*^th^ time point, $\theta_{j,k}$ is the phase of each cell at each time point, and m= 20. The half-life of the amplitude was determined from the time at which the amplitude was less than half the initial value.

### 2.7. Simulation

To simulate the circadian rhythm of a cell population, we used the Kuramoto model (Kuramoto, 1984), which represents a globally coupled oscillator population. This model is expressed by the following equation:


(4) $$\frac{d\theta_{j}}{dt}=\omega_{j}+\frac{k}{N}\overset{N}{\underset{l=1}{\sum }}\sin\left(\theta_{l}-\theta_{j}\right)+Z\left(\theta_{j}\right)LD\left(t\right)$$

where $\theta_{j}$ is the phase of the *j*th cell, and the initial phases of all cells are set to 0 (rad). N is the number of cells, and N = 1,000. $\omega_{j}$ is the frequency of individual cells and follows a normal distribution with the mean of 2π/τ and standard deviation of 2π/τ×0.1 , where τ is the free-running period: τ = 24 (h) in wild-type mice and τ = 26 (h) in mice with DSPS. k represents the coupling strength, and the simulations were performed at k = 0.01, 0.03, 0.05, and 0.1. Z(θ) is the response function to light, and Z(θ)=0.05cosθ . LD(t) represents the light conditions and is equal to 1 during the light period and 0 during the dark period. We performed simulations under DD and jet-lag conditions in wild-type mice and under LD conditions in mice with DSPS.

The synchronization rate R and the mean phase Θ of the cell population were determined using the following equation,


(5) $$Re^{i\;\Theta }=\frac{1}{N}\overset{N}{\underset{j=1}{\sum }}e^{i\theta_{j}}$$

where i is an imaginary unit and e is Euler’s number (Kuramoto, 1984). The behavioral patterns of mice are represented by sin Θ .

### 2.8. Statistics

No statistical methods were used to determine the sample size. The experiments were randomized. The investigators were not blinded to allocation during the experiments. The onsets of control mice and mice administered with 40 mg·kg^−1^·day^−1^ aripiprazole and the c-fos expression were compared using two-way ANOVA (Šídák’s multiple comparison test was also performed for the onsets). The PS50 values of different doses of aripiprazole in drinking water, the circadian period, and Qp value of mouse wheel-running behavioral rhythms in constant darkness were compared using one-way ANOVA, and multiple comparisons test for the PS50 values and circadian periods. The results are expressed as mean ± SEM with individual value plots. For experiments on the bioluminescence rhythms of SCN slice cultures, unpaired Student’s *t*-tests were used to compare each parameter between DMSO and 20 μM aripiprazole groups, except the period, which was compared using Welch’s *t*-test. All results are expressed as mean ± SEM with individual values. The synchronization rate was calculated by comparing the phase relationships of 20 randomly selected neurons in each SCN slice, as described above (section 2.6). Temporal changes in the synchronization rates of all DMSO and 20 μM aripiprazole samples throughout the first 4 days were compared using two-way ANOVA. Line graphs showing mean ± SEM at each time point were plotted to show the results. The synchronization rates of DMSO and 20 μM aripiprazole groups were compared using unpaired Student’s *t*-test, and the results are presented as mean ± SEM with individual value plots. For the cAMP assay, bioluminescence under the control and aripiprazole-treated conditions was compared using unpaired Student’s *t*-test. For the cAMP assay involving WAY-100635, the area under curve (AUC) from 50 min after aripiprazole application (as the first 50 min contain noise from drug application) to 500 min afterward for both the control and WAY-100635 applied conditions was calculated. The AUC of both groups was compared using Welch’s *t*-test. All statistical analyses were performed using GraphPad Prism.

## 3. Results

### 3.1. Chronic aripiprazole administration accelerates entrainment to external light–dark cycles

**Figure 1:**
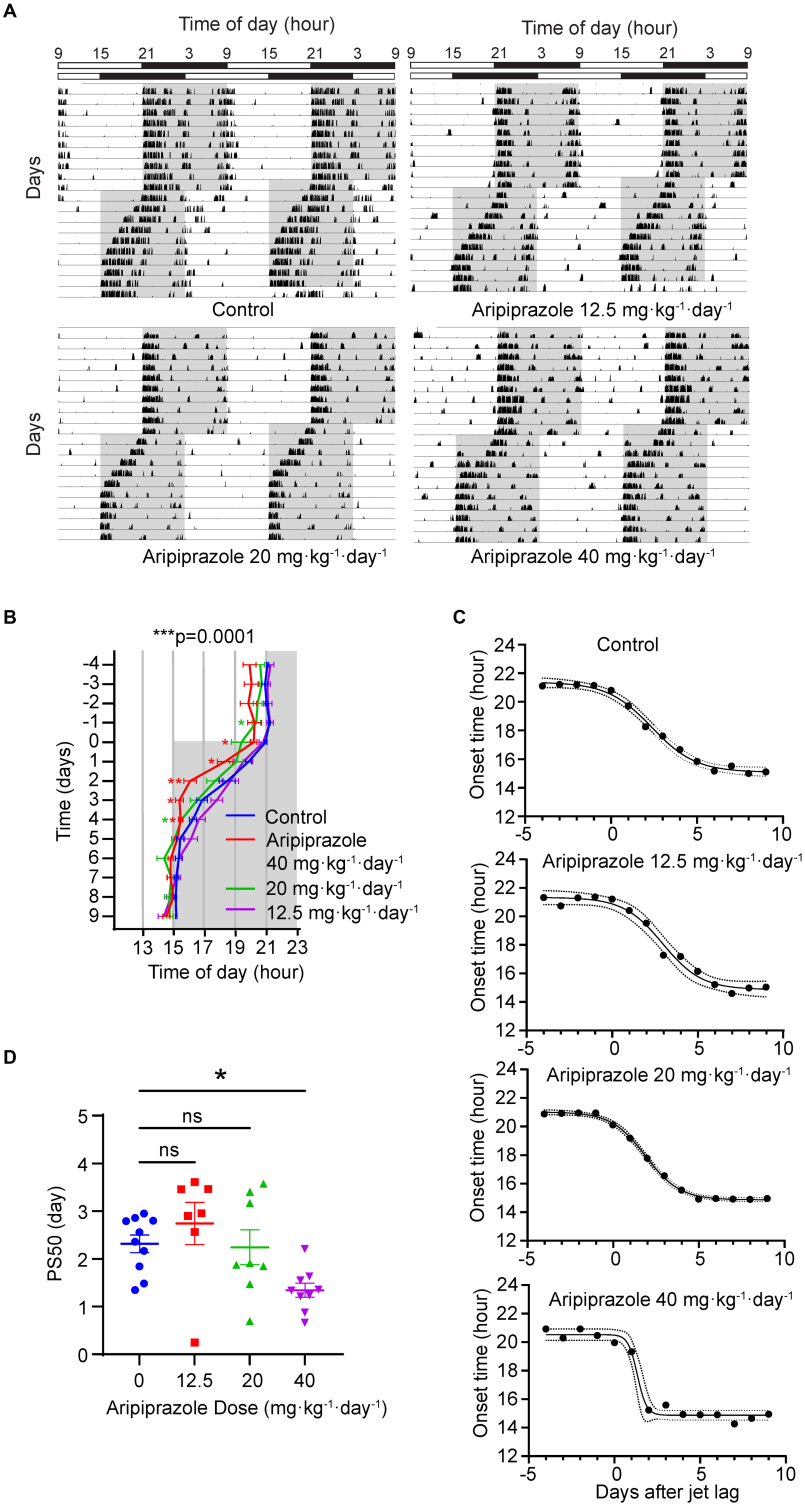
Effect of oral aripiprazole administration on re-entrainment to a 6 h advanced light–dark (LD) cycle. **(A)** Representative double-plotted actogram showing the wheel-running activity of control mice (top left) and mice administered with 12.5 (top right), 20 (bottom left), or 40 (bottom right) mg·kg^−1^·day^−1^ aripiprazole, subjected to a 6 h phase advance in LD cycles. Bar on the top shows lighting conditions. Gray area in the actogram represents the period when the lights were off. **(B)** Activity onsets 4 days before and 10 days after jet-lag. The date of phase shift of the LD cycle was set to day 0. Gray area represents the period when the lights were off. Data are presented as mean ± SEM (*n* = 10 for control, *n* = 7 for 12.5, *n* = 9 for 20, and 40 mg·kg^−1^·day^−1^ aripiprazole-administered group; ***p = 0.0001; two-way ANOVA; *p = 0.0485 for control vs. 20 mg·kg^−1^·day^−1^ aripiprazole on day −1, *p = 0.0212 for control vs. 40 mg·kg^−1^·day^−1^ aripiprazole on day 0; *p = 0.0497 for control vs. 40 mg·kg^−1^·day^−1^ aripiprazole on day 1; **p = 0.0057 for control vs. 40 mg·kg^−1^·day^−1^ aripiprazole on day 2; *p = 0.0104 for control vs. 40 mg·kg^−1^·day^−1^ aripiprazole on day 3; *p = 0.0269 for control vs. 40 mg·kg^−1^·day^−1^ aripiprazole, *p = 0.0333 for control vs. 20 mg·kg^−1^·day^−1^ aripiprazole on day 4). **(C)** Representative sigmoid dose–response curve fitting of the activity onsets of control (top), 12.5 (second from top), 20 (second from bottom), and 40 (bottom) mg·kg^−1^·day^−1^ aripiprazole-administered groups. The date of phase shift of the LD cycle was set to day 0 on the *x*-axis. Dotted line represents 95% CI. **(D)** The 50% phase shift value (PS50) of 0 (control), 12.5, 20, and 40 mg·kg^−1^·day^−1^ aripiprazole-administered groups. Data are presented as mean ± SEM (*n* = 10 for control, *n* = 7 for 12.5 mg·kg^−1^·day^−1^ aripiprazole, *n* = 8 for 20 and *n* = 9 for 40 mg·kg^−1^·day^−1^ aripiprazole; *p = 0.0125; ordinary one-way ANOVA; *p = 0.0405 for control vs. 40 mg·kg^−1^·day^−1^ aripiprazole; multiple comparisons test).

We hypothesized that aripiprazole, owing to its beneficial effects on the phenotype observed in human patients with delayed sleep phase, may ameliorate symptoms by modulating the circadian clock. Specifically, we postulated that aripiprazole may enhance the subjects’ adaptation to external light–dark cycles by directly affecting photoentrainment; this is because SCN neurons express monoamine receptors, which are involved in photoentrainment and are targets of aripiprazole. To validate this hypothesis, we chronically administered aripiprazole to wild-type mice at 0, 12.5, 20, and 40 mg·kg^−1^·day^−1^ via drinking water. We then monitored the wheel-running activity of the mice to examine the effects of the drug on photoentrainment. After over 10 days of aripiprazole administration, the mice were subjected to jet-lag (6 h phase advance) (Figure 1A). We quantified the onset time of the active period and compared the transition of onset. A significant difference in onset time was observed among the groups (*n* = 10 for control, *n* = 7 for 12.5, *n* = 9 for 20 and 40 mg·kg^−1^·day^−1^ aripiprazole-administered group; ***p = 0.0001; two-way ANOVA; *p = 0.0485 for control vs. 20 mg·kg^−1^·day^−1^ aripiprazole on day −1, *p = 0.0212 for control vs. 40 mg·kg^−1^·day^−1^ aripiprazole on day 0; *p = 0.0497 for control vs. 40 mg·kg^−1^·day^−1^ aripiprazole on day 1; **p = 0.0057 for control vs. 40 mg·kg^−1^·day^−1^ aripiprazole on day 2; *p = 0.0104 for control vs. 40 mg·kg^−1^·day^−1^ aripiprazole on day 3; *p = 0.0269 for control vs. 40 mg·kg^−1^·day^−1^ aripiprazole, *p = 0.0333 for control vs. 20 mg·kg^−1^·day^−1^ aripiprazole on day 4; Figure 1B). Further, the speed of entrainment to the new light–dark cycle was calculated using sigmoidal curve fitting to determine the PS50 value (Figure 1C). Aripiprazole treatment at 40 mg·kg^−1^·day^−1^ resulted in a significantly lower PS50 than control treatment (n = 10 for control, n = 7 for 12.5 mg·kg^−1^·day^−1^ aripiprazole, n = 8 for 20 and n = 9 for 40 mg·kg^−1^·day^−1^ aripiprazole; *p = 0.0125; ordinary one-way ANOVA; *p = 0.0405 for control vs. 40 mg·kg^−1^·day^−1^ aripiprazole; multiple comparisons test; Figure 1D). We also determined the actual dose of aripiprazole for each mouse based on their daily water consumption and observed a dose-dependency in the enhancement of entrainment to the advanced light–dark cycle (Supplementary Figure S1). To explore another possibility, we investigated whether the enhanced entrainment in jet-lag is a result of increased photosensitivity of the retina or altered input to the SCN through the retinohypothalamic tract signaling by aripiprazole. For this purpose, we compared the c-fos induction by a short light-pulse in mice administered with 40 mg·kg^−1^·day^−1^ aripiprazole or only 1% arabic gum (as control) for two weeks (Supplementary Figure S2A). We found that aripiprazole administration did not affect the level of c-fos expression regardless of light stimulation (n = 5 for each condition; *p* = 0.1789; two-way ANOVA Supplementary Figure S2B). Therefore, chronic oral administration of high-dose aripiprazole accelerates entrainment to advanced light–dark cycles in mice without affecting the photic input to the SCN.

We next subjected wild-type mice receiving aripiprazole to constant darkness and measured the free-running wheel-running activity (Supplementary Figure S3A). We calculated the power of the chi-square periodogram referred to as Qp value, which is also an indicator of rhythm robustness, and period of each group. There was no significant difference in the Qp values between each group (n = 10 for control, n = 9 for 20 and 40 mg·kg^−1^·day^−1^ aripiprazole; *p* = 0.4808; ordinary one-way ANOVA; Supplementary Figure S3B). This suggests that aripiprazole itself does not disrupt rhythmicity at the phenotypic level. Additionally, we observed diminished activity levels in mice administered with aripiprazole (Supplementary Figure S3A). This might be a secondary effect of aripiprazole that is independent of circadian regulation, such as somnolence and locomotor irregularities (Davenport et al., 2004; Kikuchi et al., 1995). Interestingly, we observed that the groups that were administered aripiprazole displayed a significantly shorter circadian period of behavioral rhythms (*n* = 10 for control, *n* = 9 for 20 and 40 mg·kg^−1^·day^−1^ aripiprazole; ***p = 0.0003; ordinary one-way ANOVA; *p = 0.0127 for control vs. 20 mg·kg^−1^·day^−1^ aripiprazole; ***p = 0.0001 for control vs. 40 mg·kg^−1^·day^−1^ aripiprazole; multiple comparisons test; Supplementary Figure S3C). We deduced that aripiprazole administration modulates the circadian clock, affecting aspects such as the clock speed and entrainment to advanced light–dark cycles in mice.

### 3.2. Aripiprazole application to SCN slice cultures disrupts cellular synchrony, thereby damping the rhythm

**Figure 2:**
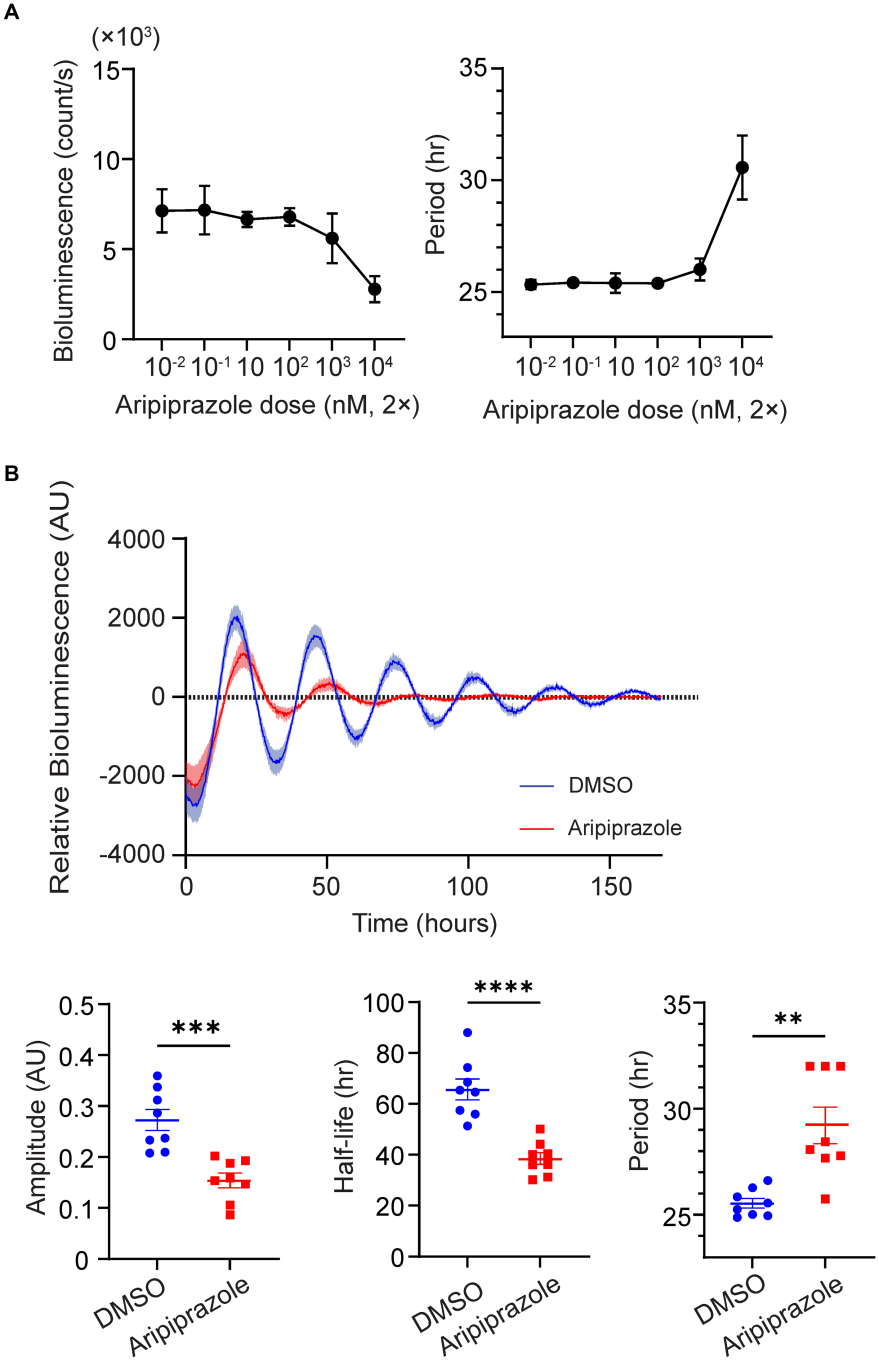
High-dose aripiprazole application decreased the rhythm amplitude and accelerated the damping of the rhythm. **(A)** Bioluminescence (left) and circadian period (right) of PER2::LUC rhythms in SCN cultures treated with different doses of aripiprazole. Data are presented as mean ± SEM (*n* = 3 for each dose). **(B)** The top graph represents detrended relative bioluminescence of SCN cultures treated with either DMSO (blue) or 20 μM aripiprazole (red). Bottom three bar graphs and plots of individual values represent the amplitude (left), half-life of amplitude (middle), and circadian period (right) of SCN cultures treated with either DMSO (blue) or 20 μM aripiprazole (red). Data are presented as mean ± SEM (*n* = 8 for DMSO and 20 μM aripiprazole; ***p = 0.0004 for comparison of amplitude; ****p < 0.0001 for comparison of half-life; unpaired Student’s *t*-test; **p = 0.0034 for comparison of period; Welch’s *t*-test).

To examine whether aripiprazole directly affects the function of the central clock, we first assessed the effect of aripiprazole on cellular rhythms by monitoring PER2::LUC bioluminescence in SCN slice cultures derived from homozygous *mPer2^Luc^* knock-in mice expressing the clock protein PER2 fused with LUC. We applied aripiprazole to SCN slice cultures at various concentrations and found that 20 μM aripiprazole tended to decrease the bioluminescence values of PER2::LUC (Figure 2A). Further, we examined the effect of 20 μM aripiprazole on different parameters of the circadian properties of PER2::LUC rhythms. Aripiprazole (20 μM) decreased the rhythm amplitude, accelerated the damping of the rhythm, and extended the circadian period (n = 8 for DMSO and 20 μM aripiprazole; ***p = 0.0004 for comparison of amplitude; ****p < 0.0001 for comparison of half-life; unpaired Student’s *t*-test; **p = 0.0034 for comparison of period; Welch’s *t*-test; Figure 2B). Although we observed a period-shortening effect of aripiprazole in the behavioral analysis (Supplementary Figure S3C), it has an opposite effect on the period of PER2::LUC rhythms in *ex vivo* slice cultures. These results suggest that aripiprazole significantly affects clock function.

**Figure 3:**
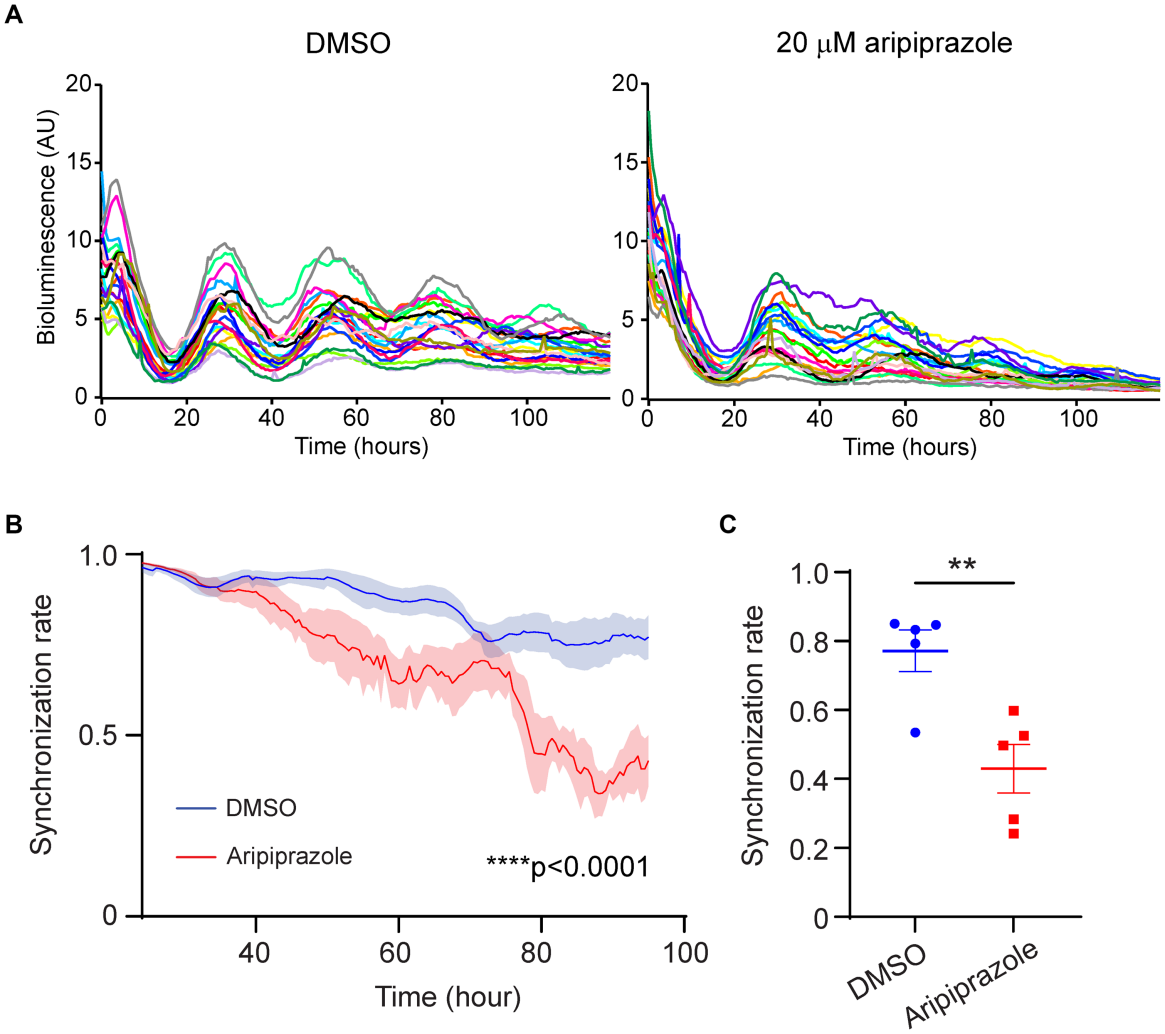
Aripiprazole application to SCN slice cultures disrupted cellular synchrony. **(A)** Representative graph showing the PER2::LUC bioluminescence of 20 neurons in SCN slices treated with DMSO (left) or 20 μM aripiprazole (right). **(B)** Temporal changes in the synchronization rate on the first 4 days in the SCN slices treated with DMSO (blue) or 20 μM aripiprazole (red). Data are presented as mean ± SEM (*n* = 5 for DMSO and 20 μM aripiprazole; ****p < 0.0001; two-way ANOVA). The synchronization rate was calculated based on the phases of 20 neurons for each slice. **(C)** The synchronization rate on day 4 after DMSO (blue) or 20 μM aripiprazole (red) application is shown. Data are presented as mean ± SEM. (*n* = 5 for DMSO and 20 μM aripiprazole; **p = 0.0060; unpaired Student’s *t*-test).

In a previous study, weakening interneuronal communication within the SCN by knocking out arginine vasopressin receptors (*V1a* and *V1b*) facilitated entrainment to external light stimuli in mice exposed to jet-lag (Yamaguchi et al., 2013). Thus, reduced cellular coupling within the SCN is one of the factors augmenting photic entrainment. To examine whether the rapid damping observed in the aripiprazole-treated SCN at the population level was due to the desynchronization of SCN neurons, we performed bioimaging on SCN slices at the single-cell level. We calculated the temporal changes in the synchronization rate in each slice based on the circadian phases of 20 randomly selected neurons (Figure 3A). Slices treated with 20 μM aripiprazole displayed a gradual decrease in the synchronization rate over the first 4 days of recording (n = 5 for DMSO and 20 μM aripiprazole; ****p < 0.0001; two-way ANOVA; Figure 3B). On day 4, the test slices displayed a significantly lower synchronization rate than the controls (*n* = 5 for DMSO and 20 μM aripiprazole; **p = 0.0060; unpaired Student’s *t*-test; Figure 3C). These observations suggest that aripiprazole disrupts SCN cellular synchrony, leading to the rapid damping of cellular rhythms at the population level.

### 3.3. Aripiprazole affects intracellular signaling in the SCN partially through the 5-HT_1A_R

**Figure 4:**
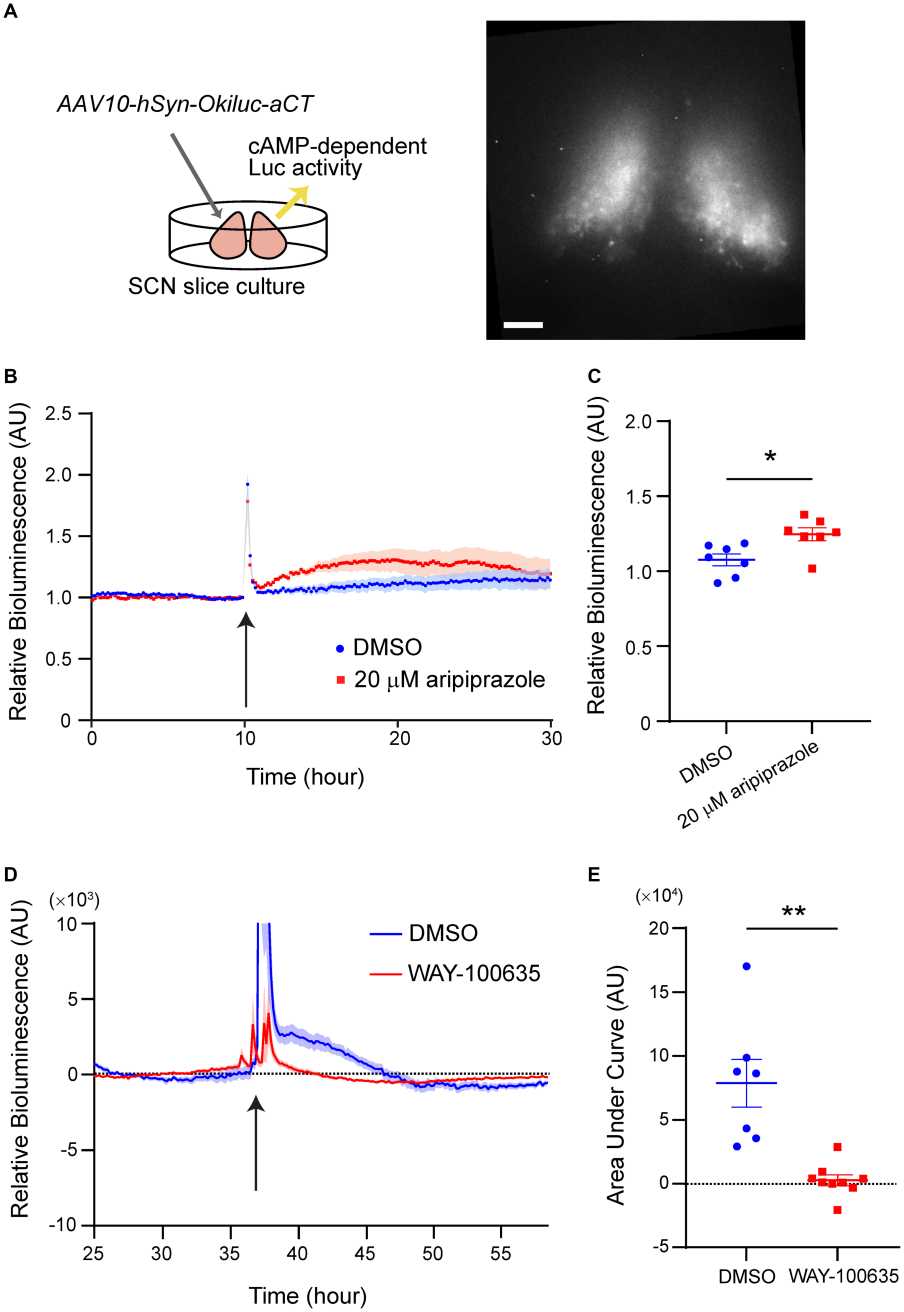
5-HT_1A_R mediated the increase of cellular cAMP level in the SCN treated with aripiprazole. **(A)** Experimental scheme of the cAMP assay (left) and representative bioluminescence image acquired with Cellgraph (right). Scale bar = 100 μm. **(B)** Relative bioluminescence of Okiluc-aCT in SCN slices treated with DMSO (blue) or 20 μM aripiprazole (red). Arrow represents the time of drug application. Data are presented as mean ± SEM. **(C)** Normalized relative bioluminescence 5 h after DMSO (blue) or 20 μM aripiprazole (red) administration is shown. Data are presented as mean ± SEM (*n* = 7 for DMSO and 20 μM aripiprazole; *p = 0.0130; unpaired Student’s *t*-test). **(D)** Detrended relative bioluminescence of Okiluc-aCT in SCN slices treated with DMSO (blue) or 20 μM WAY-100635 (red) prior to application of 20 μM aripiprazole. Arrow represents the time of aripiprazole application. Data are presented as mean ± SEM. **(E)** Area under the curve (AUC) from 50 min after aripiprazole application to 500 min afterward was calculated for both groups (*n* = 7 for DMSO and *n* = 9 for WAY-100635; **p = 0.0059; Welch’s *t*-test).

Since aripiprazole is known to act on multiple GPCRs, including those that couple to the Gi or Gs subclasses of G proteins, we examined whether the effect of aripiprazole on SCN neurons mobilized the intracellular signaling of Gi/s proteins by performing a cAMP assay. SCN slices were prepared from wild-type mice and a recently developed cAMP bioluminescence sensor, Okiluc-aCT (Ono et al., 2023), was expressed using a viral vector (Figure 4A). Okiluc-aCT expression was confirmed based on bioluminescence imaging. Bioluminescence signals were predominantly localized in the SCN (Figure 4A). We monitored the bioluminescence of Okiluc-aCT for 24 h and applied either 20 μM aripiprazole or DMSO to the culture. The slices treated with aripiprazole exhibited an increase in intracellular cAMP levels following drug application compared with the control slices (Figure 4B). When the cAMP level 5 h after drug application was compared between the two groups, the group receiving aripiprazole displayed significantly elevated cAMP levels (*n* = 7 for DMSO and 20 μM aripiprazole; *p = 0.0130; unpaired Student’s *t*-test; Figure 4C). These results suggest that aripiprazole increases intracellular cAMP levels in the SCN.

We next zeroed in on the receptor in the SCN that mediates this phenomenon. 5-HT_1A_R is a Gi-coupled receptor expressed in the SCN and is involved in photic entrainment (Moriya et al., 1998; Takahashi et al., 2002). Therefore, we tested whether 5-HT_1A_R plays a role in mediating the cAMP increase observed in the previous experiment. We similarly performed cAMP assay under the condition of pre-treatment with WAY-100635, an antagonist of 5-HT_1A_R, 24-h before administering aripiprazole to the culture medium. The cultures that received only DMSO before aripiprazole application displayed an increase in cAMP levels by aripiprazole administration, similar to the previous experiment (Figure 4D). Pre-treatment with WAY-100635 impaired the increase in cAMP levels resulting from aripiprazole application (Figure 4D). Cultures treated with only DMSO prior to aripiprazole application displayed significantly higher cAMP level after aripiprazole administration compared to the cultures that received WAY-100635 (*n* = 7 for DMSO and n = 9 for WAY-100635; **p = 0.0059; Welch’s *t*-test; Figure 4E). These results suggest that 5-HT_1A_R plays a significant role in aripiprazole-mediated cAMP signaling pathway in the SCN.

## 4. Discussion

Previous studies on human participants have indicated that patients diagnosed with certain psychiatric disorders accompanied by sleep disorders who received aripiprazole as part of treatment displayed improved daily sleep rhythms (Takaki and Ujike, 2014; Matsui et al., 2017; Tashiro, 2017; Omori et al., 2018; Imanishi et al., 2021; Konishi et al., 2022). Prior to aripiprazole administration, many patients exhibited delayed sleep onset and sleep patterns similar to those of free-running rhythms (Matsui et al., 2017; Tashiro, 2017). However, following aripiprazole treatment, these patients could fall asleep at more normal times, typically from midnight to early morning (Matsui et al., 2017; Tashiro, 2017).

In the present study, we examined the effect of aripiprazole on mouse circadian behavior and demonstrated that oral aripiprazole administration in mice significantly accelerated entrainment during jet-lag experiments (6 h advanced light–dark cycle; Figure 1). Additionally, the application of aripiprazole to slice cultures of the SCN desynchronized SCN neurons, thereby attenuating the amplitude of PER2::LUC oscillation and accelerating the dampening of the rhythm at the population level (Figures 2, 3). Therefore, we deduced that aripiprazole facilitates faster entrainment to external cues through desynchronization of SCN neurons. Similarly, our simulations demonstrated that reduced cellular coupling among SCN neurons was associated with an increased response to a shifted light–dark cycle (Supplementary Figures S4A,B), which is consistent with previous reports in *V1a-* and *V1b*-knockout mice (Yamaguchi et al., 2013). Of note, our simulations further demonstrated that a mouse model of DSPS with a 26-h free-running period would display a free-running rhythm even under a 12/12 h light–dark cycle when the coupling between neurons was strong, but would entrain to the external light–dark cycle if the coupling was weak (Supplementary Figure S4C). Thus, the effects of aripiprazole observed in human patients with DSPS may be attributed to the desynchronization of SCN neurons and increased response to external photic stimuli, which enable a more facile adjustment to environmental light–dark cycles. We also observed shortening of period in mice administered with aripiprazole (Supplementary Figures S3A,C). This could be another factor contributing to the advancement of sleep onset observed in patients with delayed sleep phase receiving aripiprazole. However, in the *ex vivo* slice experiment, we observed lengthening of period from aripiprazole addition (Figure 2B). The period elongation seen in the *ex vivo* slices administered with aripiprazole could be due to a different mechanism from the shortened period observed at the behavioral level. We also confirmed that aripiprazole does not directly modulate the photic input to the SCN from the retina (Supplementary Figure S2). While it did not reach statistical significance, we found that aripiprazole administration slightly increases c-fos positivity compared to the control mice regardless of whether there was light stimulation. We speculate that this is due to the direct effect of aripiprazole on the basal activity of the SCN neurons.

Aripiprazole exhibits a prominent effect on circadian behavior and SCN cellular rhythms; however, the mechanism of action remains to be elucidated owing to its complexity. Aripiprazole possesses different affinities and exhibits diverse responses to multiple monoamine receptors (Shapiro et al., 2003), and its agonistic or antagonistic effects depend on the drug/ligand concentration as well as the expression levels of the target receptors. Since the SCN expresses various monoamine receptors, aripiprazole may induce differential effects on multiple receptors with varying affinities, which may account for the phenomena observed in the present study. Previous studies have shown that the activities of dopamine and serotonin receptors affect photic entrainment (Kennaway and Moyer, 1998; Moriya et al., 1998; Takahashi et al., 2002; Varcoe et al., 2003; Varcoe and Kennaway, 2008; Westrich et al., 2013; Grippo et al., 2017). The activation of 5-HT_1A_R, a Gi-coupled receptor, by a selective full agonist significantly accelerated entrainment to new light–dark cycles in mice (Moriya et al., 1998; Takahashi et al., 2002). There are also reports that the combination of a selective 5-HT_7_ receptor antagonist, SB269970, and a selective serotonin reuptake inhibitor, escitalopram, extended the period in *ex vivo* SCN slice cultures (Westrich et al., 2013). Furthermore, the *in vivo* administration of this combination induced robust phase delays. 5-HT_2C_R is a Gq-coupled serotonin receptor expressed in the SCN (Pickard and Rea, 1997) that is known to induce core clock gene expression in the SCN and also phase shifts various physiological rhythms, such as melatonin and core body temperature rhythms (Kennaway and Moyer, 1998; Varcoe et al., 2003; Varcoe and Kennaway, 2008).

In the present study, using a cAMP indicator, we observed that aripiprazole increased cAMP signaling in SCN slice cultures (Figure 4). Since cAMP signaling is involved in SCN cellular coupling (O’Neill et al., 2008), disturbances in cAMP signaling mediated by aripiprazole may have resulted in the desynchronization of SCN neurons. The slight increase in c-fos expression through aripiprazole administration (Supplementary Figure S2) may be caused by the aripiprazole acting through a monoamine receptor in the SCN that involves the cAMP signaling pathway. We also found that pre-treatment with an antagonist of 5-HT_1A_R, WAY-100635, inhibited the increase in cAMP levels induced by aripiprazole application, deducing that the 5-HT_1A_R is one of the receptors responsible for the effects of aripiprazole. But since aripiprazole targets a vast number of receptors, many of which are expressed in the SCN, it is likely that 5-HT_1A_R is not the only receptor involved in the phenomena we observed in mouse behavior and SCN rhythms. Further studies on calcium signaling would address the involvement of other receptors such as 5-HT_2C_R in the effect of aripiprazole. It is also still unclear if the targeting of 5-HT_1A_R in the SCN by aripiprazole is the direct cause of SCN cellular desynchrony.

Aripiprazole has been widely applied clinically as an atypical antipsychotic drug to treat depression, bipolar disorder, and schizophrenia; however, growing evidence suggests that it has other functions in improving sleep disorders (Takaki and Ujike, 2014; Matsui et al., 2017; Omori et al., 2018; Imanishi et al., 2021). In the present study, we clarified that aripiprazole modulates the master clock in the SCN, which in turn alters circadian function, resulting in a faster adaptation to phase shift from 6-h advanced jet-lag in mice. Furthermore, the improved photic entrainment observed after the jet-lag procedure in mice treated with aripiprazole suggests that the drug may be beneficial to alleviate jet-lag or circadian dysfunction caused by shift work. Our findings shed light on the potential physiological role of aripiprazole and opens new avenues for its future clinical applications.

## Data availability statement

The original contributions presented in the study are included in the article/Supplementary material, further inquiries can be directed to the corresponding authors.

## Ethics statement

The animal studies were approved by Seiya Mizuno, Institute of Medicine, University of Tsukuba. The studies were conducted in accordance with the local legislation and institutional requirements. Written informed consent was obtained from the owners for the participation of their animals in this study.

## Author contributions

RL, KM, DO, AH, and TS: methodology. AH, TK, and TS: funding acquisition. AH and TS: project administration. AH and TS: supervision. RL, KM, TK, AH, and TS: writing. All authors contributed to the article and approved the submitted version.

## Funding

This work was supported by Japan Agency for Medical Research and Development (AMED) (grant numbers JP19dm0908001, JP20dm0107162, and JP21zf0127005 to TK; grant number JP21zf0127005 to TS; and grant numbers JP21zf0127003 and JP22gm6410030 to AH), Japan Society for the Promotion of Science (JSPS) KAKENHI (grant number JP19K22465 to TS), Japan Science and Technology Agency (JST) CREST (grant number JPMJCR1655 to TS), JSPS KAKENHI Grant-in- Aid for Scientific Research [(C):19 K08037 and 22K07571 to TK], The Naito Foundation (AH), TMFC Japan Foundation for applied Enzymology (AH), and JSPS Grant-in-Aid for JSPS Fellows (grant number 21 J20226 to RL).

## Conflict of interest

The authors declare that the research was conducted in the absence of any commercial or financial relationships that could be construed as a potential conflict of interest.

## Publisher’s note

All claims expressed in this article are solely those of the authors and do not necessarily represent those of their affiliated organizations, or those of the publisher, the editors and the reviewers. Any product that may be evaluated in this article, or claim that may be made by its manufacturer, is not guaranteed or endorsed by the publisher.

## Footnotes

^1^ https://lmfit.github.io/lmfit-py/
