# Supplementary material for: Aripiprazole disrupts cellular synchrony in the suprachiasmatic nucleus and enhances entrainment to environmental light–dark cycles in mice
Source: Front Neurosci. 2023 Aug 9;17:1201137. doi: 10.3389/fnins.2023.1201137 (PMC10445652; doi:10.3389/fnins.2023.1201137)
Supplement: Supplementary file 1 [file Data_Sheet_1.docx]

Supplementary Material

# Supplementary Figures


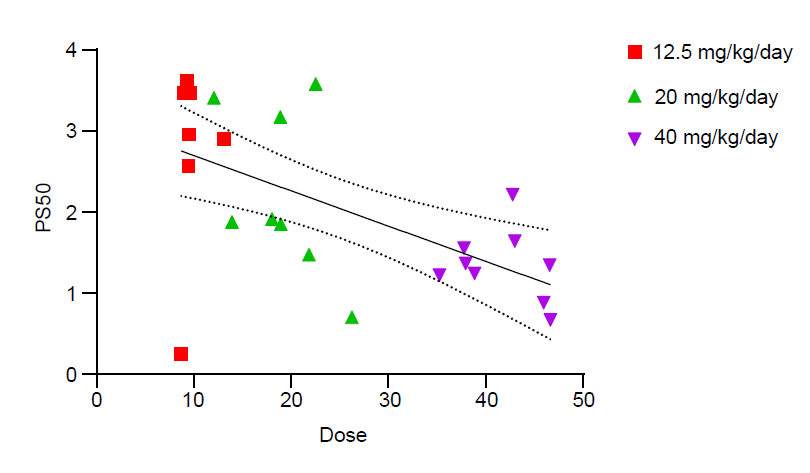


**Supplementary Figure 1**

The correlation between actual intake amount of aripiprazole and PS50 of each mouse. Full line represents the linear trend, dotted line represents 95% CI (**p=0.0022; r=-0.5951: Pearson’s correlation).


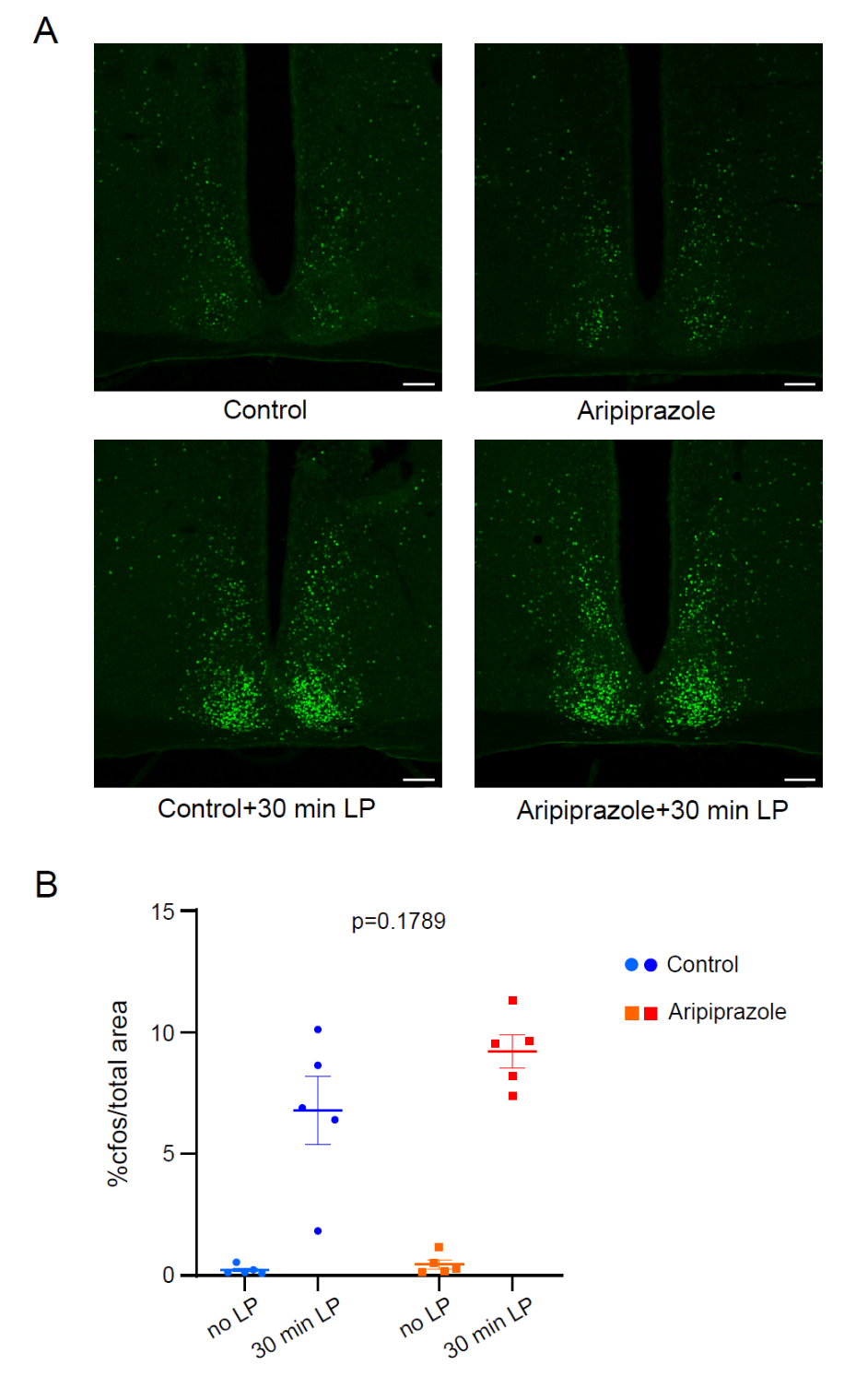


**Supplementary Figure 2**

Aripiprazole administration does not affect photic induction of c-fos in the SCN. (A) Representative images of coronal slices of SCN and surrounding areas obtained with confocal microscope. C-fos expression was visualized by immunohistochemistry using anti-c-fos antibody. Control mice and mice administered with 40 mg·kg^-1^·day^-1^ aripiprazole were sacrificed at ZT22 without a light pulse (top) or with a 30 min light pulse (LP) from ZT21.5 (bottom). Scale bar= 100 μm. (B) The percentage of c-fos positive areas in the SCN of control mice and mice administered with 40 mg·kg^-1^·day^-1^ aripiprazole was calculated. LP= light pulse. Data are represented as mean±SEM (n=5 for each condition; p= 0.1789; two-way ANOVA).


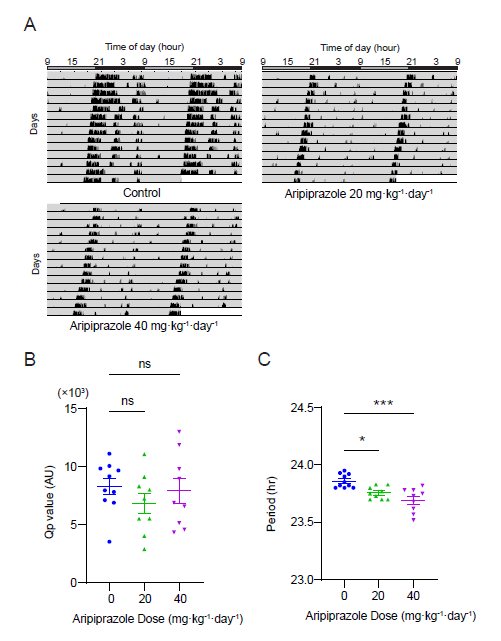


**Supplementary Figure 3**

Effect of oral aripiprazole administration on free-running rhythm in mice. (A) Representative double-plotted actogram showing the wheel-running activity of control mice (top left) and mice administered with 20 (top right) or 40 (bottom left) mg·kg^-1^·day^-1^ aripiprazole in constant darkness. Gray area in the actogram represents the period when the lights were off. (B) The Qp value (indicator of rhythm robustness) of 0 (control), 20, and 40 mg·kg^-1^·day^-1^ aripiprazole-administered groups. Data are presented as mean±SEM (n=10 for control, n=9 for 20 and 40 mg·kg^-1^·day^-1^ aripiprazole; p=0.4808; ordinary one-way ANOVA). (C) The circadian period of 0 (control), 20, and 40 mg·kg^-1^·day^-1^ aripiprazole-administered groups. Data are presented as mean±SEM (n=10 for control, n=9 for 20 and 40 mg·kg^-1^·day^-1^ aripiprazole; ***p=0.0003; ordinary one-way ANOVA; *p=0.0127 for control vs. 20 mg·kg^-1^·day^-1^ aripiprazole; ***p=0.0001 for control vs. 40 mg·kg^-1^·day^-1^ aripiprazole; multiple comparisons test).

**Supplementary Figure 4**

Simulations of the effect of different degrees of coupling strength between SCN neurons on the entrainment rate. (A) Changes in the synchronization rate among SCN neurons with various coupling strengths under constant conditions in simulation. *k* represents the coupling strength. (B, C) Simulation of the entrainment of behavioral rhythms to 6 h advanced jet-lag in wild-type mice (B) and entrainment to light–dark cycles in DSPS model mice (C) exhibiting various coupling strengths. SCN coupling strengths are indicated below the simulated actograms. *k* represents the coupling strength. Solid and dashed lines represent the transitions from light to dark and from dark to light, respectively. The value of sinΘ represents the behavioral pattern of mice. Red and blue areas indicate resting and active periods, respectively.
